# ﻿*Selaginelladensiciliata* (subg. *Heterostachys*, Selaginellaceae), a new spikemoss species from China based on morphological and molecular data

**DOI:** 10.3897/phytokeys.227.101222

**Published:** 2023-06-07

**Authors:** Shao-Li Fang, Bo Xu, Liang Zhang, Zhao-Rong He, Xin-Mao Zhou

**Affiliations:** 1 School of Ecology and Environmental Science & School of life Sciences, Yunnan University, Kunming, 650500, Yunnan, China Yunnan University Kunming China; 2 CAS Key Laboratory of Mountain Ecological Restoration and Bioresource Utilization and Ecological Restoration and Biodiversity Conservation Key Laboratory of Sichuan Province, Chengdu Institute of Biology, Chinese Academy of Sciences, Chengdu, China Chengdu Institute of Biology, Chinese Academy of Sciences Chengdu China; 3 CAS Key Laboratory for Plant Diversity and Biogeography of East Asia, Kunming Institute of Botany, Chinese Academy of Sciences (CAS), Kunming 650201, Yunnan, China Kunming Institute of Botany, Chinese Academy of Sciences Kunming China

**Keywords:** Medog, *Selaginellavaginata*, submonomorphic sporophylls, *S.* subg. *Heterostachys*

## Abstract

A new species of spikemoss, *Selaginelladensiciliata* in S.subg.Heterostachyssect.Tetragonostachyae, China, is described from southeastern Xizang, based on morphological and molecular phylogenetic data. Morphologically, *S.densiciliata* is similar to *S.repanda*, *S.subvaginata* and *S.vaginata*, but the new species can be easily distinguished from them by having sterile leaves margins densely ciliate, symmetrical axillary leaves oblong ovate to ovate-triangular, and ovate dorsal leaves obviously carinate. Molecular phylogenetic analysis resolves *S.densiciliata* as sister to the clade comprised with *S.vaginata* and *S.xipholepis*, which confirms the recognition of the new species.

## ﻿Introduction

Located in southeastern Xizang, Medog county and adjacent regions are one of the biodiversity hotspots in the world (Myers et al. 2000; Mittermeier et al. 2005), even harboring the highest species diversity of plants in China (Sun and Zhou 1996). According to Du et al. (2021), the most number of new species of plants have been discovered in Medog, among all the counties in China in 2020. From 2015 to 2022, most of the authors (Bo Xu, Liang Zhang, Xin-Mao Zhou and Zhao-Rong He) carried out several field investigations and collected a large number of specimens in Medog. Based on those collections, three fern species, *Athyriumaberrans* Liang Zhang & Li Bing Zhang, *Hymenaspleniumtholiformis* Liang Zhang, W.B. Ju & K.W. Xu, and *Selligueawusugongii* Liang Zhang, X.P.Fan & Li Bing Zhang have been discovered (Fan et al. 2021; Qiu et al. 2022a, b). When we studied the lycophytes from these collections, we found some materials of *Selaginella* belonging to the *S.vaginata* group, but differing from all recognized species in this group.

The *Selaginellavaginata* group, including at least three species, i.e., *S.subvaginata* X.C.Zhang & Shalimov, *S.repanda* (Desv. & Poir.) Spring, and *S.vaginata* Spring, represents a taxonomically difficult group in S.sect.Tetragonostachyae of S.subg.Heterostachys sensu Zhou and Zhang (2015). Another classification with seven subgenera in *Selaginella* was also proposed by Weststrand and Korall (2016a). However, a subsequent study has evidenced that *Stachygynandrum* Weststrand & Korall (2016a) (~600 species) was not monophyletic (Zhou et al. 2022). In this study, Zhou and Zhang (2015)’s classification was followed. The *Selaginellavaginata* group is characterized by generally small plants, nearly monomorphic sporophylls, and more or less ciliate margins of leaves (Zhang et al. 2013).

Our previous phylogenetic study of *Selaginella* firstly found that the *S.vaginata* (= *S.compta* Hand.-Mazz. in Zhou et al. 2016) group was not monophyletic and it clustered with those species with distinctly dimorphic sporophylls (e.g., *S.albociliata* P.S.Wang, *S.ciliaris* (Retz.) Spring, *S.lutchuensis* Koidz, *S.xipholepis* Baker) in the *S.ciliaris* clade (= “Asia” clade in Zhou et al. 2016) of S.sect.Tetragonostachyae (Zhou and Zhang 2015; Zhou et al. 2016). Also, *S.vaginata* (= *S.compta*) was paraphyletic in relation to *S.xipholepis* (Zhou et al. 2016). Subsequently, Zhang et al. (2020) confirmed the non-monophyly of the *S.vaginata* group and described a new species, *S.subvaginata* X.C. Zhang & Shalimovin. S*elaginella vaginata* is widely distributed in East, Southeast and South Asia, and its elevations range from 500 to 3,600 m according to Zhang et al. (2013) and our own field investigation.

Our further studies of the morphology, phylogeny, and spore morphology of those species related to the *S.vaginata* group confirm that materials from Medog represent a new species. We describe it here as *Selaginelladensiciliata*.

## ﻿Materials and methods

### ﻿Morphological study

Field observations were conducted in June (in 2015) and October (in 2017) respectively. The photos of plants, leaves, and strobili were taken in the field. All research materials were deposited at KUN and PYU (Index Herbarium: Thiers 2018). More details of morphology were observed and photographs were taken using SMZ1270 stereo microscope (Nikon, Japan). Megaspores and microspores were selected and attached to Carbon Adhesive Tape (CAT) using anatomical lens, then samples were coated with gold using the BAL-TEC SCD 005 Cool Sputter Coater (BAL-TEC AG., Liechtenstein) and visualized via QUANTA 200 Scanning Electron Microscope (SEM) (FEI Co., USA) at 25 kV at Yunnan University, Kunming, China. The morphological terminology of spore follows Tryon and Lugardon (1991) and Zhou et al. (2015).

### ﻿DNA extraction, amplification and sequencing

Total genomic DNA of seven samples (one from *Selaginelladianzhongensis* X.C.Zhang, two from *S.wuyishanensis* K.W.Xu, X.M.Zhou & Y.F.Duan, and four from the new species) was extracted from silica-dried material using the TIANGEN plant genomic DNA extraction kit (TIANGEN Biotech., Beijing, China) following the manufacturers’ protocols. One nuclear locus (ITS) and one plastid gene (rbcL) were selected for amplification and sequencing. Primers and the PCR conditions followed Zhou et al. (2016). Amplified fragments were purified with TIANquick Mini Purification Kits (Tiangen Biotech, Beijing, China) and purified polymerase chain reaction (PCR) products were sequenced by Tsingke (Kunming, China). Fourteen sequences were newly generated in this study (7 5.8S+ITS2 and 7 *rbcL*) (Appendix 1). Newly generated sequences were edited and assembled using Sequencher v. 4.1.2 (Gene Codes Corporation, Ann Arbor, Michigan).

### ﻿Phylogenetic analysis

Based on a previous phylogenetic study of *Selaginella* (Zhou et al. 2016; Weststrand and Korall 2016b), three species, *S.bisulcata* Spring, *S.nipponica* Franch. & Sav., and *S.uncinata* (Desv. ex Poiret) Spring from subg. Heterostachys sensu Zhou and Zhang (2015) were selected as outgroups. A total 102 accessions representing 58 species of *Selaginella* were sampled (Appendix 1). All sequences were aligned using MAFFT ver. 7 (Katoh and Standley 2013), followed by manual adjustment in BioEdit (Hall 1999). A matrix with 1702 characters (5.8S + ITS2: 425 and *rbcL*: 1277) was used for phylogeny study. The jModeltest2 (Darriba et al. 2012) was used to choose the best-fitting likelihood model. The AIC (Akaike information criterion) was used to select the best model (Akaike 1974), GTR+I+G, was chosen for the Maximum likelihood (ML) and Bayesian inference (BI) analysis for combined dataset. Maximum likelihood (ML) bootstrapping was performed with 1000 rapid bootstrap replicates (BS) analyses followed by a search for the best-scoring tree in a single run in RAxML v. 8 (Stamatakis et al. 2008). Bayesian inference (BI) was conducted using MrBayes ver. 3.2.7a (Ronquist and Huelsenbeck 2003) with two runs of four Markov chain Monte Carlo (MCMC) chains, each beginning with a random tree and sampling every 1000 generations for 10,000,000 generations. Convergence among runs and stationarity was assessed using Tracer ver. 1.4 (Rambaut and Drummond 2007), and the first 25% was discarded as burnin. The remaining trees were used to calculate a 50% majority-rule consensus topology and posterior probabilities (PP). ML and BI analyses were executed on Cipres (Miller et al. 2010).

## ﻿Results and discussion

The aligned length of combined plastid gene (*rbcL*: 1277 bp) and nuclear loci (ITS: 425 bp) was 1702 bp, of which 1242 sites were identical, 389 characters were parsimony informative, and 71 variable characters were parsimony-uninformative.

**Figure 1.**
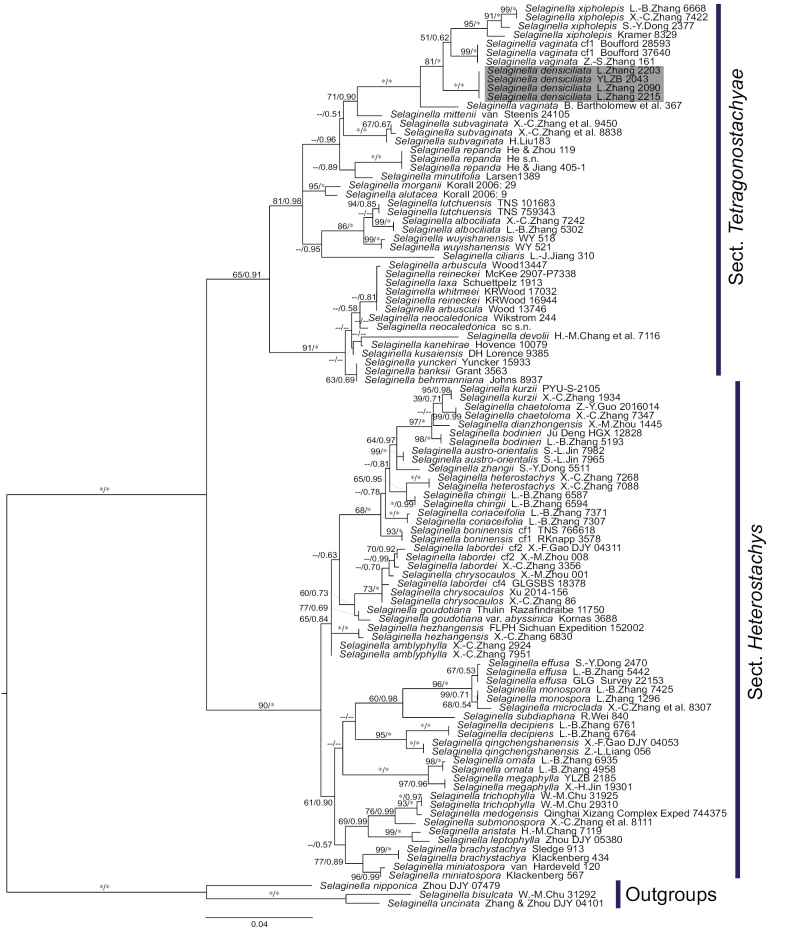
Maximum likelihood phylogeny of *Selaginelladensiciliata* and its allies in subg. Heterostachys. based on molecular data. The numbers associated with branches are maximum likelihood bootstrap support (MLBS) ≥ 50% and Bayesian posterior probability (BIPP) ≥ 0.50; the dash (--) indicates MLBS < 50% or BIPP < 0.50; the asterisk indicates MLBS = 100 or BIPP = 1.00; omitted support values indicate both MLBS < 50% and BIPP < 0.50. Sections followed Zhou and Zhang (2015)’s classification.

As our previous phylogenetic studies for *Selaginella* (Zhou et al. 2016, 2022), phylogeny showed that S.sect.Heterostachys (Baker) Li Bing Zhang & X.M.Zhou is sister to S.sect.Tetragonostachyae (Fig. 1). The trees from the ML and BI analyses revealed identical topologies, and four samples of the new species form a highly supported clade (MLBS=100; BIPP=1.00, Fig. 1). *Selaginelladensiciliata* is sister to a clade containing some samples of *S.vaginata* and all samples of *S.xipholepis*. *Selaginelladensiciliata* is a distant relative of its morphologically similar species (*S.repanda*, *S.subvaginata* and *S.vaginata*). As previous studies (e.g., Zhou et al. 2016; Zhang et al. 2020) have suggested, both the *S.vaginata* group (including *S.repanda*, *S.subvaginata* and *S.vaginata*) and *S.vaginata* itself are not monophyletic (Fig. 1) and two lineages were found. With extensive sampling, potentially, more new taxa will be detected and evidenced in the *S.vaginata* group.

**Figure 2.**
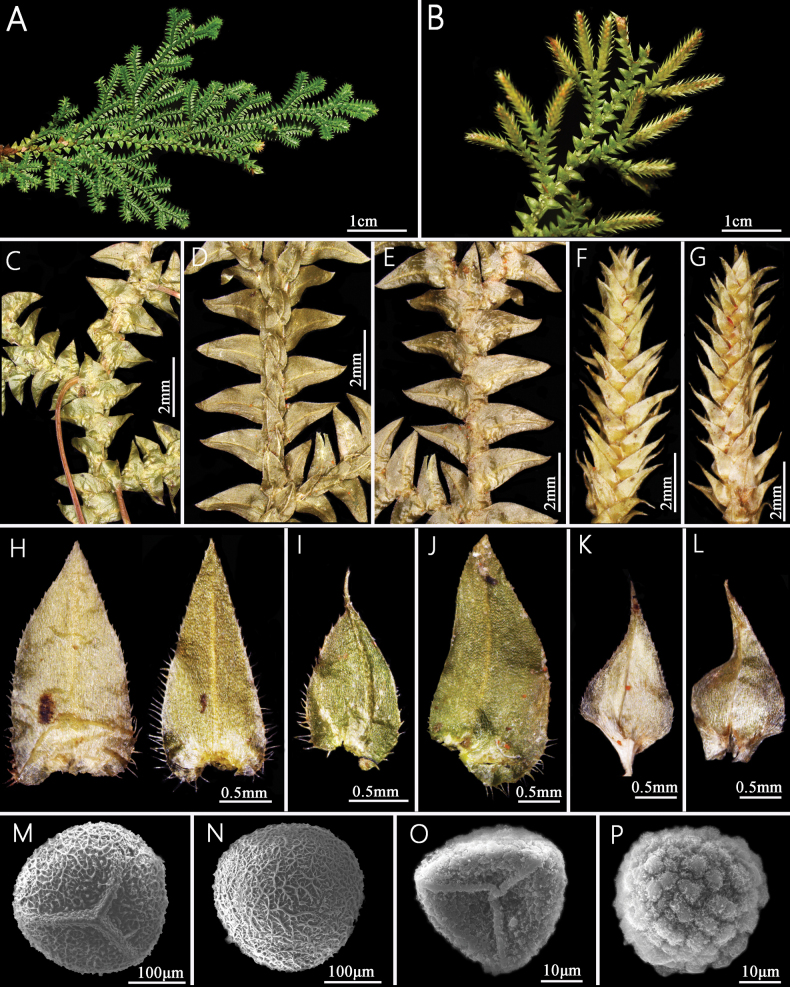
*Selaginelladensiciliata***A** dorsal view of branches **B** ventral view of branches with strobili **C** ventral view of branches, showing rhizophores, axillary leaves, and ventral leaves **D** dorsal view of branches, showing dorsal leaves **E** ventral view of branches, showing axillary leaves, and ventral leaves **F** dorsal view of strobilus **G** ventral view of strobilus **H** axillary leaf on stems (left) and branches (right) **I** dorsal leaf **J** ventral leaf **K** dorsal sporophyll **L** ventral sporophyll **M** proximal surface of megaspore **N** distal surface of megaspore **O** proximal surface of microspore **P** distal surface of microspore (from the holotype: *L. Zhang* et al. *2215*).

Comparison of morphological characters between *Selaginelladensiciliata* and its morphologically similar species is shown in Table 1. *Selaginelladensiciliata* is easily distinguished from other species in the *S.vaginata* morphological group by sterile leaves with margins densely ciliate at the lower parts (or at least at base) (Fig. 2H–J), ventral leaves falcate (Fig. 2C, E, J), dorsal leaves obvious carinate (Fig. 2D, I), axillary leaves symmetrical (Fig. 2C, E, H), and fine reticulation of megaspore surfaces (Fig. 2M, N).

**Table 1.** Morphological comparison among *Selaginelladensiciliata*, *S.repanda*, *S.subvaginata*, and *S.vaginata*.

	*Selaginelladensiciliata*	*S.repanda*	*S.subvaginata*	*S.vaginata*
Habit	Ascending to suberect	Suberect or ascending	Suberect	Creeping
Leaves margin	Not white-margined	White-margined	Not white-margined	White-margined
Axillary leave	Symmetrical, oblong-ovate to ovate-triangular	Symmetrical, ovate-lanceolate	Asymmetrical, ovate to ovate-triangular	Asymmetrical, ovate-triangular
Dorsal leave	Ovate, margins ciliate, apex aristate; carinate	Obliquely ovate, margins denticulate; slightly carinate	Ovate-lanceolate, inner margins ciliate; slightly carinate	Ovate-lanceolate or ovate-triangular, base margins ciliate; slightly carinate
Ventral leave	Oblong-falcate, basiscopic base margins denticulate	Ovate or obliquely ovate, basiscopic base margins ciliate	Oblong-falcate, basiscopic base margins denticulate	Ovate-lanceolate or ovate, basiscopic base margins denticulate
Dorsal sporophyll	Ovate, margins denticulate	Ovate-lanceolate, margins denticulate	Ovate, base margins ciliate	Ovate-lanceolate, base margins ciliate
Ventral sporophyll	Broadly ovate, margins denticulate	Broadly ovate, margins denticulate	Ovate, base margins ciliate	Ovate-lanceolate, margins denticulate
Megaspore	Fine reticulate	Verrucate	Fine reticulate	Verrucate
Microspore	Verructae and rugulate with spiny microstructure	Verructae and rugulate	Smooth	Verructae and rugulate

Submonomorphic sporophylls are similar dorsal and ventral sporophylls in morphology, but dorsal sporophylls are slightly larger than ventral ones. Submonomorphic sporophylls are only present in some species of S.sect.Heterostachys (e.g., *S.monospora* Spring) and S.sect.Tetragonostachyae (Hook. & Grev.) Hieron. & Sadeb. in S.subg.Heterostachys sensu Zhou and Zhang (2015). Submonomorphic sporophylls are derived from distinctly dimorphic sporophylls in S.subg.Heterostachys Baker sensu Zhou and Zhang (2015) (Fig. 1, Zhou et al. 2016).

### ﻿Taxonomic treatment

#### ﻿ Selaginella densiciliata

Taxon classificationPlantaeSelaginellalesSelaginellaceae

X.M.Zhou, Liang Zhang & Bo Xu sp. nov.

B2761967-8FDB-5D36-BA91-5DC6E1EE8106

urn:lsid:ipni.org:names:77320720-1

##### Type.

China. Xizang: Medog County, Beibeng Township, on the way from A’niqiao to #3 bridge, in broad-leaved evergreen forest, 29°20'41.56"N, 95°9'56.99"E, elev. 1600 m, 15 Oct. 2017, *Liang Zhang*, *Wen-Bin Ju & Heng-Ning Deng2215* (holotype: KUN-1572683!, isotypes: KUN-1572684!, PYU-02074721!, PYU-02074722!).

##### Diagnosis.

**Figure 3.**
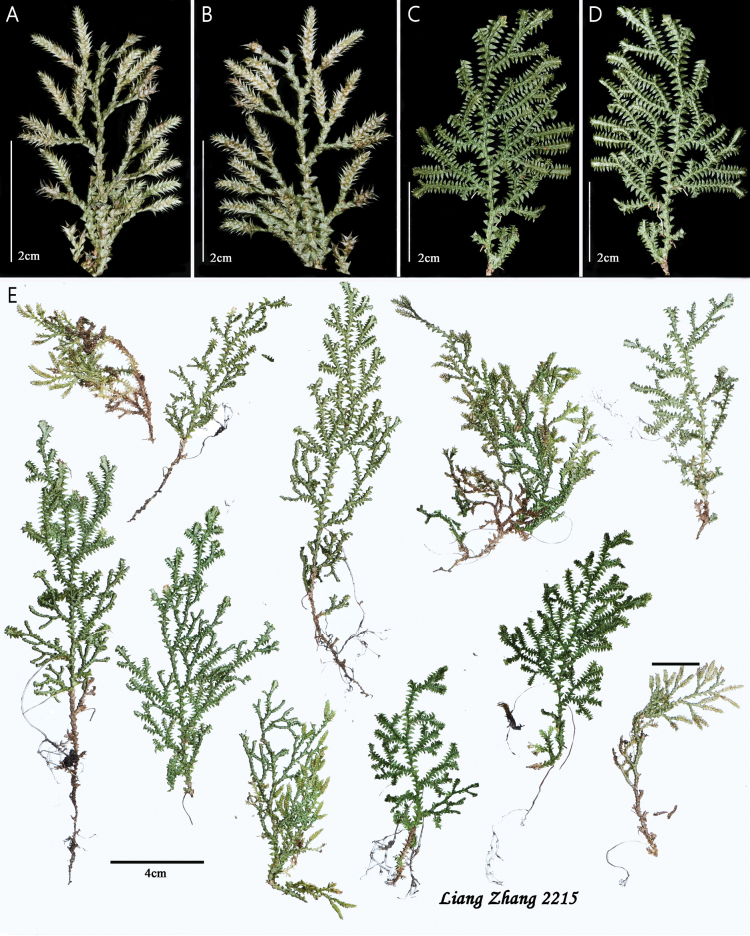
Type of *Selaginelladensiciliata***A** dorsal view of branches with strobili **B** ventral view of branches with strobili **C** dorsal view of branches **D** ventral view of branches **E** holotype of *Selaginelladensiciliata* (*L. Zhang* et al. *2215*).

*Selaginelladensiciliata* is similar to *S.repanda*, *S.subvaginata*, and *S.vaginata* in having relatively small plants (Fig. 3E), base of stem with ventral leaves strongly curly and surrounding stem when dry (Fig. 3E), submonomorphic sporophylls (Fig. 2B, F, G), but the new species has sterile leaves margins densely ciliate at base (Fig. 2H–J), dorsal leaves obviously carinate (Fig. 2D, I), symmetrical axillary leaves oblong-ovate ovate-triangular (Fig. 2H), and megaspore surfaces fine reticulate (Fig. 2M, N).

##### Description.

***Plants*** terrestrial, evergreen, ascending to suberect, 7.0–15.0 cm tall, without creeping rhizomes or stolons, without elongate tuber at base of stem (Fig. 3E). ***Rhizophores*** grow from the base to the middle of stem, borne on ventral side in axils of branches (Figs 2C, 3E), 0.2–0.3 mm in diam. ***Main stem*** branched upwards from near the base, pinnately branched (Figs 2A, 3), unbranched main stem is 0.5–3.5 cm tall, terete, glabrous, 0.4–0.6 mm in diam (Fig. 3). ***Branches*** 6–14 pairs, 2 or 3 times pinnately branched; adjacent main branches on main stem 3.1–5.0 mm apart, the terminal branches 2.6–5.2 mm wide (Fig. 3C–E). ***Sterile leaves*** four rows, leathery, margins densely ciliate on the lower parts or at least at base (Fig. 2H–J). ***Axillary leaves*** on main stems not larger than those on branches, symmetrical, oblong-ovate to ovate-triangular ovate-triangular, base not peltate, truncate; axillary leaves on branches symmetrical, oblong-ovate to ovate-triangular, base cordate, 1.6–2.1×0.8–1.2 mm, slightly carinate, margins densely ciliate in basal half to denticulate at apex, apex acute (Fig. 2C, E, H). ***Dorsal leaves*** asymmetrical, those on main stems strongly larger than those on branches; ovate, 1.2–1.9 × 0.5–1.0 mm, strongly carinate, base truncate, oblique, peltate, margins slightly densely ciliate at basal half, upward denticulate, apex shortly aristate (Fig. 2C, E, J). ***Ventral leaves*** asymmetrical, overlapping stem and branches, those on main stem strongly larger than those on branches, oblong-falcate, 2.0–2.7×1.0–1.5 mm, carinate, base round, peltate, apex acute; basiscopic margins slightly denticulate at base, upward subentire; acroscopic base margins densely ciliate at lower part, upward subentire (Fig. 2D, I). ***Strobili*** solitary, terminal, compact, quadrangular, 4.3–7.9 mm (Figs 2B, F, G, 3A, B). ***Sporophylls*** slightly dimorphic, dorsal sporophylls slightly longer than ventral sporophylls (Fig. 2B, F, G); dorsal sporophylls ovate, carinate, 1.4–1.5×0.7–0.9 mm, margins denticulate, base cuneate, not peltate, apex acuminate, without sporophyll-pteryx (Fig. 2K); ventral sporophylls broadly ovate, carinate, 1.23–1.47×0.69–0.81 mm, base truncate, not peltate, apex acuminate, margin denticulate (Fig. 2G). Megasporophylls in basal portion on lower side of strobilus. ***Megaspore*** white-yellow, oblate spheroid to subglobose, 225.6–280.2 μm in diam., prominent laesurae extend 2/3 of the distance to the equator; surface finely reticulate ornamentation (Fig. 2M, N). ***Microspore*** orange, hemispherical, 27.4–37.7 μm, surfaces with dense and large verrucate ornamentation covered with densely irregular granular microstructure (Fig. 2O, P).

##### Geographical distribution and habitat.

*Selaginelladensiciliata* is only known from Beibeng Township, Medog County, Xizang Province, China. It grows in humid places in evergreen broadleaved forests, at elevations of 1000–1600 m.

##### Additional specimens examined (paratypes).

**China. Xizang: Nyingchi City, Medog county, Beibeng township.** on the way from A’niqiao to Hanmi village, elev. ca. 1000 m, 29°20'14.40"N, 95°10'19.19"E, 4 Jun. 2015. *Bo Xu & Xin-Mao Zhou YLZB2043* (CDBI, PYU); on the way from A’niqiao to Hanmi village, elev. 1530 m, 29°20'29.51"N, 95°10'12.74"E, 15 Oct. 2017, *Liang Zhang*, *Wen-Bin Ju & Heng-Ning Deng 2090* (KUN, PYU); on the way from A’niqiao to Hanmi village, alt. 1120 m, 29°19'42.75"N, 95°10'36.47"E, 17 Oct. 2017, *Liang Zhang*, *Wen-Bin Ju & Heng-Ning Deng 2203* (KUN, PYU).

##### Etymology.

The specific epithet “*densiciliata*” is a compound word derived from the Latin word “*dense*” which means dense and suffix “*ciliata*” which means ciliate. The specific epithet “*densiciliata*” refers to sterile leave (axillary leaves, dorsal leaves, and ventral leaves, Fig. 2H–J) margins with dense cilia at base.

### ﻿Key to *Selaginelladensiciliata* and its relative species of the *S.vaginata* group

**Table:** 

1	Stem nearly creeping, only fertile parts (strobili) ascending	***S.vaginata***
–	Stem more or less suberect or ascending	**2**
2	Leaves distinctly white-margined	***S.repanda***
–	Leaves not obviously white-margined	**3**
3	Base of sterile leave margins sparsely ciliate or denticulate; axillary leaves asymmetrical; dorsal leaves ovate-lanceolate, slightly carinate; microspore surface smooth	***S.subvaginata***
–	Base of sterile leave margins densely ciliate; axillary leaves symmetrical; dorsal leaves ovate, obvious carinate; microspore surface verrucate	***S.densiciliata***

## Supplementary Material

XML Treatment for Selaginella densiciliata

## ﻿Additional information

### Data availability

All of the data that support the findings of this study are available in the main text or Supplementary Information.

## ﻿Appendix 1

List of taxa sampled with information related to taxonomy, GenBank accession numbers (*rbcL*, ITS), references, and vouchers information. Herbarium codes follow Index Herbariorum (Thiers 2018).

***S.albociliata* P. S. Wang** (1) *L.-B. Zhang* et al.et al. 5302 (CDBI), China (Guangxi), KT161379 (Zhou et al. 2016), KT161648 (Zhou et al. 2016); (2) *X.-C. Zhang 7242* (PE), China (Guizhou), MH814882 (Shalimov et al. 2019), —. ***S.alutacea* Spring***Korall 2006-9* (S), Malaysia, KY022958 (Weststrand and Korall 2016b), —. ***S.amblyphylla* Alston** (1) *X.-C. Zhang 2924* (PE), China (Yunnan), MH814883 (Shalimov et al. 2019), —; (2) *X.-C. Zhang 7951* (PE), China (Yunnan), MH814884 (Shalimov et al. 2019), —. ***S.arbuscula* Spring** (1) *Wood 13447* (PTBG), Hawaii (Kauai, Wainiha), KT161387 (Zhou et al. 2016), KT161656 (Zhou et al. 2016); (2) *Wood 13746* (PTBG), Hawaii (Maui, Kipahulu), KT161388 (Zhou et al. 2016), KT161657 (Zhou et al. 2016). ***S.aristata* Spring***H.-M. Chang* et al. *7119* (TAIE), MF313959 (Sheue et al. Unpublished), —. ***S.austro*-*orientulis* H. J. Wei & X. M. Zhou** (1) *S.-L. Jin* et al. *7965* (CSH, IBK, PYU), China (Jiangxi), OP690605 (Wei et al. 2023), OP683200 (Wei et al. 2023); (2) *S.-L. Jin* et al. *7982* (CSH), China (Jiangxi), OP690606 (Wei et al. 2023), OP683199 (Wei et al. 2023). ***S.banksii* Alston***Grant 3563* (L), French Polynesia, KY022972 (Weststrand and Korall 2016b), —. ***S.behrmanniana* Hieron***Johns 8937* (L), Indonesia, KY022973 (Weststrand and Korall 2016b), —. ***S.bisulcata* Spring***Chu* et al. *31292* (PYU), China (Yunnan), KT161404 (Zhou et al. 2016), KT161673 (Zhou et al. 2016). ***S.bodinieri* Hieron.** (1) *Ju & Deng HGX12828* (CDBI), China (Sichuan), KT161414 (Zhou et al. 2016), KT161677 (Zhou et al. 2016); (2) *L.-B. Zhang* et al. *5193* (CDBI), China (Guangxi), KT161409 (Zhou et al. 2016), KT161680 (Zhou et al. 2016). ***S.boninensis* Baker.** (1) *Knapp 3578* (P), China (Taiwan), MZ571146 (He et al. 2021), —; (2) *TNS766618* (TNS), Japan (Tokyo), AB574642 (Ebihara et al. 2010), —. ***S.brachystachya* (Hook. & Grev.) Spring** (1) *J. Klackenberg 434* (S), Sri Lanka, KY022980 (Weststrand and Korall 2016b), —; (2) *W.A. Sledge 913* (L), Sri Lanka, KY022979 (Weststrand and Korall 2016b), —. ***S.chaetoloma* Alston** (1) *Z.-Y. Guo 2016014* (PE), China (Guizhou), MH814888 (Shalimov et al. 2019), —; (2) *X.-C. Zhang 7347* (PE), China (Guizhou), MH814889 (Shalimov et al. 2019), —. ***S.chingii* Alston** (1) *L.-B. Zhang* et al. *6587* (CDBI, MO, VNMN, PYU), Vietnam (Lang Son), KT161417 (Zhou et al. 2016), KT161683 (Zhou et al. 2016); (2) *L.-B. Zhang* et al. *6594* (CDBI, MO, VNMN, PYU), Vietnam (Lang Son), KT161416 (Zhou et al. 2016), KT161868 (Zhou et al. 2016). ***S.chrysocaulos* (Hook. & Grev.) Spring** (1) *Xu* et al. *2014-156* (CDBI), China (Sichuan), KT161427 (Zhou et al. 2016), KT161690 (Zhou et al. 2016); (2) *X.-M. Zhou 001* (CDBI), China (Yunnan), MZ532020 (He et al. 2021), —; (3) *X.-C. Zhang 86* (PE), China (Sichuan), MH814891 (Shalimov et al. 2019), —. ***S.ciliaris* (Retz.) Spring***Jiang 310* (PYU, CDBI), China (Hainan), KT161428 (Zhou et al. 2016), KT161691 (Zhou et al. 2016). ***S.coriaceifolia* X. M. Zhou, N. T. Lu & Li Bing Zhang** (1) *L.-B. Zhang* et al. *7307* (CDBI, MO, VNMN), Vietnam (Quang Binh), MT386596 (He et al. 2021), MZ570596 (He et al. 2021); (2) *L.-B. Zhang* et al. *7371* (CDBI, MO, VNMN), Vietnam (Quang Binh), MT386598 (Ye et al. 2020), MT386595 (Ye et al. 2020). ***S.decipiens* Warb.** (1) *L.-B. Zhang* et al. *6761* (CDBI, MO, VNMN, PYU), Vietnam (Bac Kan), KT161439 (Zhou et al. 2016), KT161697 (Zhou et al. 2016); (2) *L.-B. Zhang* et al. *6764* (CDBI, MO, VNMN, PYU), Vietnam (Bac Kan), KT161438 (Zhou et al. 2016), KT161698 (Zhou et al. 2016). ***S.densiciliata* Xin-Mao Zhou & Liang Zhang** (1) *L. Zhang* et al. *2090* (KUN, PYU), China (Xizang), OQ723681 (this study), OQ728789 (this study); (2) *L. Zhang* et al. *2203* (KUN, PYU), China (Xizang), OQ723684 (this study), OQ728792 (this study); (3) *L. Zhang* et al. *2215* (KUN, PYU), China (Xizang), OQ723682 (this study), OQ728790 (this study); (4) *B. Xu & X.-M. Zhou 2043* (CDBI, PYU), China (Xizang), OQ723683 (this study), OQ728791 (this study). ***S.devolii* H. M. Chang***H.-M. Chang* et al. *7116* (TAIE), Unknown, MF313957 (Sheue et al. 2017), —. ***S.dianzhongensis* X. C. Zhang***X.-M. Zhou 1445* (PYU), China (Yunnan), OQ723685 (this study), OQ728793 (this study). ***S.effusa* Alston** (1) *Dong 2470* (PYU), China (Guangdong), (Zhou et al. 2016), KT161705 (Zhou et al. 2016); (2) *GLG Survey 22153* (GH), China, KY023020 (Weststrand and Korall 2016b), —; (3) *L.-B. Zhang* et al. *5442* (CDBI), China (Guangxi), KT161451 (Zhou et al. 2016), KT161707 (Zhou et al. 2016). ***S.goudotiana* Spring***M. Thulin and H. Razafindraibe 11750* (UPS), Madagascar, KY023039 (Weststrand and Korall 2016b), —. **S.goudotianaSpringvar.abyssinica (Spring) Bizzarri***J. Kornaś and A. MedweckaKornaś 3688* (BR), Zamb, KY023036 (Weststrand and Korall 2016b), —. ***S.heterostachys* Baker** (1) *X.-C. Zhang 7088* (PE), China (Guizhou), MH814896 (Shalimov et al. 2019), —; (2) *X.-C. Zhang 7268* (PE), China (Guizhou), MH814897 (Shalimov et al. 2019), —. ***S.hezhangensis* P. S. Wang et X. Y. Wang** (1) *FLPH Sichuan Expedition 152002*, China (Sichuan), OM864654 (Shalimov and Zhang 2022), —; (2) *X.-C. Zhang 6830* (PE), China (Guizhou), OM864656 (Shalimov and Zhang 2022), —. ***S.kanehirae* Alston** (1) *Hovence 10079* (PTBG), Hawaii, MZ571148 (He et al. 2021), —. ***S.kurzii* Baker** (1) *X.-M. Zhou* et al. *PYU-S-2105* (PYU), China (Yunnan), MZ532022 (He et al. 2021), MZ570598 (He et al. 2021); (2) *X.-C. Zhang 1934* (PE), Unknown, MH814898 (Shalimov et al. 2019), —. ***S.kusaiensis* Hosok.***D.H. Lorence 9385* (PTBG), Unknown, MT657911 (Nitta et al. 2020), —. ***S.labordei* Hieron. ex Christ** (1) *Gao* et al. *DJY04311* (CDBI), China (Sichuan), KT161503 (Zhou et al. 2016), KT161751 (Zhou et al. 2016); (2) *X.-M. Zhou 008* (CDBI), China (Sichuan), KT161505 (Zhou et al. 2016), —; (3) *Gaoligong Shan Biodiversity Survey 18378* (GH), China (Yunnan), KY023061 (Weststrand and Korall 2016b), —; (4) *X.-C. Zhang 3356* (PE), China (Hubei), MH814899 (Shalimov et al. 2019), —. ***S.laxa* Spring** (1) *Schuettpelz 1913* (US), French Polynesia (Marquesas Islands), MT216111 (He et al. 2021), —. ***S.leptophylla* Baker***X.-M. Zhou* et al. *DJY05380* (CDBI), China (Sichuan), KT161513 (Zhou et al. 2016), KT161756 (Zhou et al. 2016). ***S.lutchuensis* Koidz.** (1) *TNS101683* (TNS), Japan, MT680176 (Zhang et al. 2020), —; (2) *TNS759343* (TNS), Japan (Okinawa), AB574648 (Ebihara et al. 2010), —. ***S.medogensis* Ching et S. K. Wu***Qinghai-Xizang Complex Exped 74-4375* (PE), China (Xizang), OK247696 (Shalimov and Zhang 2021), —. ***S.megaphylla* Baker** (1) *X.-H. Jin 19301* (PE), Unknown, MH814901 (Shalimov et al. 2019), —; (2) *X.- M. Zhou YLZB2185* (CDBI, PYU), China (Xizang), ON994456 (Xu et al. 2022), ON994203 (Xu et al. 2022). ***S.microclada* Baker***X.-C. Zhang* et al. *8307* (PE), China (Yunnan), OK247702 (Shalimov and Zhang 2021), —. ***S.miniatospora* (Dalzell) Baker** (1) *J. Klackenberg and R. Lundin 567* (S), India (Kerala), KY023081 (Weststrand and Korall 2016b), —; (2) *C. van Hardeveld and H. H. van der Werff 120* (U), India (Tamil Nadu), KY023080 (Weststrand and Korall 2016b), —. ***S.minutifolia* Spring***Larsen* et al. *1389* (S), Thailand, KY023082 (Weststrand and Korall 2016b), —. ***S.mittenii* Baker***van Steenis 24105* (L), South Africa, KY023083 (Weststrand and Korall 2016b), —. ***S.monospora* Spring** (1) *L.-B. Zhang* et al. *7425* (CDBI), Vietnam (Quang Binh), MZ571145 (He et al. 2021), —; (2) *L. Zhang 1296*, China (Yunnan), MZ532023 (Xu et al. 2022), —. ***S.morganii* Zeiller***P. Korall 2006*-29 (S), Peninsular Malaysia, KY023088 (Weststrand and Korall 2016b), —. ***S.neocaledonica* Baker** (1) *sc s.n.* Unknown, KY985453 (Klaus et al. 2017), —; (2) *Wikstrom 244* (S), New Caledonia, KY023095 (Weststrand and Korall 2016b), —. ***S.nipponica* Franch. & Sav.** (1) *Zhou* et al. *DJY07479* (CDBI), China (Sichuan), KT161542 (Zhou et al. 2016), KT161784 (Zhou et al. 2016). ***S.ornata* Spring** (1) *Zhang 4958* (CDBI), China (Guizhou), KT161522 (Zhou et al. 2016), KT161768 (Zhou et al. 2016); (2) *Zhang* et al. *6935* (CDBI, MO, VNMN, PYU), Vietnam (Ha Giang), KT161526 (Zhou et al. 2016), KT161772 (Zhou et al. 2016). ***S.qingchengshanensis* Li Bing Zhang & X. M. Zhou** (1) *Gao* et al. *DJY04053* (CDBI), China (Sichuan), KT161381 (Zhou et al. 2016), KT161649 (Zhou et al. 2016); (2) *Z.-L. Liang & X. Pu 056* (CDBI, PYU), China (Sichuan), MZ532027 (He et al. 2021), MZ570603 (He et al. 2021). ***S.reineckei* Hieron.** (1) *K.R. Wood 16944* (PTBG), Samoa (Savaii), MT657902 (Nitta et al. 2020), —; (2) *McKee 2907 - P7338* (L), Samoa, KY023129 (Weststrand and Korall 2016b), —. ***S.repanda* (Desv. ex Poir.) Spring** (1) *He & Jiang 405-1* (CDBI), China (Yunnan), KT161584 (Zhou et al. 2016), —; (2) *Z.-R. He & X.-M. Zhou 119* (PYU, CDBI), China (Yunnan), KT161583 (Zhou et al. 2016), KT161816 (Zhou et al. 2016); (3) *He s.n.* China (Yunnan), KT161817 (Zhou et al. 2016). ***S.subdiaphana* (Wall. ex Hook. et Grev.) Spring** (1) *Y.-D.Wu 840* (PE), China (Yunnan), OM864659 (Shalimov and Zhang 2022), —. ***S.submonospora* Shalimov et X.-C. Zhang***X.-C. Zhang* et al. *8111* (PE), China (Yunnan), OM864660 (Shalimov and Zhang 2022), —. ***S.subvaginata* Shalimov et X. C. Zhang** (1) *H. Liu 183* (PE), China (Sichuan), MT680178 (Zhang et al. 2020), —; (2) *X.-C. Zhang* et al. *8838* (PE), China (Sichuan), MT680179 (Zhang et al. 2020), —; (3) *X.-C. Zhang* et al. *9450* (PE), China (Sichuan), MT680181 (Zhang et al. 2020), —. ***S.trichophylla* K. H. Shing** (1) *W.-M. Chu* et al. *29310* (PYU), China (Yunnan), KT161622 (Zhou et al. 2016), KT161846 (Zhou et al. 2016); (2) *Chu* et al. *31925* (PYU), China (Yunnan), KT161621 (Zhou et al. 2016), KT161847 (Zhou et al. 2016). ***S.uncinata* (Desv. ex Poir.) Spring***Zhang & Zhou DJY04101* (CDBI), China (Sichuan), KT161626 (Zhou et al. 2016), KT161852 (Zhou et al. 2016). ***S.vaginata* Spring** (1) D.*E. Boufford* et al. *28593* (A), China (Sichuan), KY023167 (Weststrand and Korall 2016b), —; (2) *D.E. Bouff ord 37640* (A), China (Gansu), KY023168, (Weststrand and Korall 2016b), —; (3) *B. Bartholomew* et al. *367* (PE), China (Guizhou), MT680182 (Zhang et al. 2020), —; (4) *Z.-S. Zhang 161* (PE), China (Shanxi), MH814907 (Shalimov et al. 2019), —. ***S.whitmeei* Baker***K.R. Wood 17032* (PTBG), Samoa (Savaii), MT657910 (Nitta et al. 2020), —. ***S.wuyishanensis* K. W. Xu, X. M. Zhou & Y. F. Duan** (1) *K.-W. Xu WY518* (NF), China (Fujian), OQ723687 (this study), OQ728795 (this study); (2) *K.-W. Xu WY521* (NF), China (Fujian), OQ723686 (this study), OQ728794 (this study). ***S.xipholepis* Baker** (1) *S.-Y. Dong 2377* (PYU), China (Guangdong), KT161645 (Zhou et al. 2016), —; (2) *K.U. Kramer* et al. *8329* (U), China (Hong Kong), KY023179 (Weststrand and Korall 2016b), —; (3) *L.-B. Zhang* et al. *6668* (CDBI, MO, VNMN, PYU), Vietnam (Bac Kan), KT161646 (Zhou et al. 2016), KT161867 (Zhou et al. 2016); (4) *X.-C. Zhang 7422* (PE), China (Guizhou), MH814908 (Shalimov et al. 2019), —. ***S.yunckeri* Alston***Yuncker 15933* (U), Tonga, KY023182 (Weststrand and Korall 2016b), —. ***S.zhangii*S. Y. Dong***Dong 5511*, China (Yunnan), MW316869 (Huang et al. 2022), —.
